# Adult Stem Cell as New Advanced Therapy for Experimental Neuropathic Pain Treatment

**DOI:** 10.1155/2014/470983

**Published:** 2014-08-13

**Authors:** Silvia Franchi, Mara Castelli, Giada Amodeo, Stefania Niada, Daniela Ferrari, Angelo Vescovi, Anna Teresa Brini, Alberto Emilio Panerai, Paola Sacerdote

**Affiliations:** ^1^Dipartimento di Scienze Farmacologiche e Biomolecolari, Università degli Studi Milano, 20129 Milano, Italy; ^2^I.R.C.C.S. Istituto Ortopedico Galeazzi, 20161 Milano, Italy; ^3^Dipartimento di Scienze Biomediche, Chirurgiche ed Odontoiatriche, Università degli Studi di Milano, 20129 Milano, Italy; ^4^Dipartimento di Biotecnologie e Bioscienze, Università Milano Bicocca, 20126 Milano, Italy; ^5^Istituto di Ricovero e Cura a Carattere Scientifico Opera di San Pio da Pietralcina, 71013 San Giovanni Rotondo, Italy

## Abstract

Neuropathic pain (NP) is a highly invalidating disease resulting as consequence of a lesion or disease affecting the somatosensory system. All the pharmacological treatments today in use give a long lasting pain relief only in a limited percentage of patients before pain reappears making NP an incurable disease. New approaches are therefore needed and research is testing stem cell usage. Several papers have been written on experimental neuropathic pain treatment using stem cells of different origin and species to treat experimental NP. The original idea was based on the capacity of stem cell to offer a totipotent cellular source for replacing injured neural cells and for delivering trophic factors to lesion site; soon the researchers agreed that the capacity of stem cells to contrast NP was not dependent upon their regenerative effect but was mostly linked to a bidirectional interaction between the stem cell and damaged microenvironment resident cells. In this paper we review the preclinical studies produced in the last years assessing the effects induced by several stem cells in different models of neuropathic pain. The overall positive results obtained on pain remission by using stem cells that are safe, of easy isolation, and which may allow an autologous transplant in patients may be encouraging for moving from bench to bedside, although there are several issues that still need to be solved.

## 1. Introduction

Neuropathic pain (NP), currently defined as “pain arising as a direct consequence of a lesion or disease affecting the somatosensory system” [1], represents the most severe form of chronic pain considering its capacity to affect both physical and mental patient's condition. The nature of NP is extremely heterogeneous and four main categories of neuropathic lesions have been recognized: focal or multifocal lesions of the* peripheral* nervous system (PNS), lesions of the central nervous system (CNS), polyneuropathies, and complex neuropathic disorders [2]. Regardless of the primary etiology, NP can present itself as spontaneous pain sensations such as paroxysmal pain (shooting pain) and superficial pain (burning sensation) or as evoked pain: mechanical/thermal allodynia (pain caused by normally nonpainful mechanical or thermal stimuli), hyperalgesia (increased sensitivity to a normally painful stimulus), or temporal summation (increasing pain sensation from repetitive application of the identical stimulus) [3]. It has recently been pointed out that neuropathic pain pathogenesis and maintenance involve interactions among neurons, inflammatory immune cells, glial cells, and a wide cascade of pro- and anti-inflammatory cytokines [4–7]. One of the main problems concerning NP regards its scarce response to the conventional analgesic therapy. Drugs, mainly represented by tricyclic antidepressant, calcium channel ligands, SSNRI, and opioids, are in fact not fully effective and their efficacy decreases over time with development of tolerance in long term use [8, 9]. It is therefore mandatory to identify and propose novel approaches to NP treatment that could overcome many of the limitations of the available strategies.

In the last years many researchers, including us, have tried to relieve neuropathic pain by using stem cells of different origin. The first moving idea was based on the capacity of stem cell to offer a multipotent cellular source for replacing injured or lost neural cells and for delivering trophic factors to lesion site; in this way, stem cells can represent not only a pain treatment but a way for repairing the damaged nervous system at the basis of NP development. Soon we and others realized that the capacity of stem cells to contrast experimental neuropathic pain was not completely dependent upon their regenerative effect; in fact many research papers described an antinociceptive effect of the stem cell achieved before the appearance of regenerative effect [10]. In this paper we review the literature in which stem cells of different origin and species were used to treat neuropathic pain induced in experimental animal models. We divide the published papers according to the type of stem cell used, independently of the experimental NP model. We do not report the studies with embryonic stem cells considering the associated ethical problem and the major risk of tumors correlated to them. Moreover, we considered only papers in which the effect of stem cells on pain behaviour has been specifically evaluated. Today there are three main types of stem cells used for neuropathic pain: neural stem cells, mesenchymal stem cells, and bone marrow mononuclear cells.

## 2. Neural Stem Cells

Considering the nature of the lesion at the basis of NP development that takes place in PNS or CNS, neural stem cells (NSCs) seem to be the most appropriate type of cells to prompt a physiological repair of the lesion, due to their capacity to differentiate into neurons, astrocytes, and oligodendrocytes, even though it was suggested that also mesenchymal stem cells, under particular conditions, can originate cells of the neural lineage [11–13]. Neural stem cells were identified for the first time and isolated from the subventricular zone of adult mammalian brain in 1992 [14, 15]. They are multipotential precursors that grow and self-renew in culture for an extensive period of time as neurospheres, while retaining a stable capacity to generate mature functional brain cells. So far, NSC lines have been derived from the hippocampal dentate gyrus, the olfactory bulb, the SVZ surrounding the ventricles, the subcallosal zone underlying the corpus callosum, and the spinal cord of the embryonic, neonatal, and adult rodent CNS [15], as well as from human fetal CNS [16–18].

Our group [10] described for the first time the use of intravenous murine neural stem cells, NSCs, to treat neuropathic pain which develops as consequence of a lesion of the peripheral nervous system, that is, sciatic nerve chronic constriction injury (CCI). Cells, isolated from the subventricular zone using the neurosphere technique [19], were treated to express GFP gene thus allowing their localization after transplant. Even though efficiency of the transplant is low, we described the rapid and specific homing of NSC to the injured nerve, since these cells were present at lesion site starting from day 1 to day 7 after injection. Their short time presence at lesion site was, however, able to start a cascade of events in the main sites of pain transmission, which contributed to pain reduction.

Regarding their effects on pain relief, NSC, injected when the pathology was already established, induced a significant reduction in allodynia and hyperalgesia already 3 days after administration, demonstrating a therapeutic effect that lasted for at least 28 days. Responses changed with the number of administered NSCs and the effect on hyperalgesia could be boosted by a new NSC administration. Treatment induced changes in cytokine profile at lesion site, decreasing significantly the proinflammatory cytokine Interleukin-1 both as mRNA and protein, while cells were unable to normalize the levels of the anti-inflammatory cytokine IL-10 decreased by CCI. The effect on pain relief was also demonstrated by a reduction of spinal cord Fos expression in laminae I–VI. Moreover we observed a reparative process and an improvement of nerve morphology, due to NSC treatment, which was present at a later time, when pain was already controlled by NSC treatment. Since NSC effect on pain symptoms preceded nerve repair and was maintained after cell disappearance from the lesion site, we believe that the regenerative, behavioral, and immune NSC effects are largely due to microenvironmental changes that they might induce in the lesion. Our results support the idea of a general bystander effect exerted by transplanted NSC [20]. These positive results on neuropathic pain relief were supported by Xu and colleagues [21] by using another route for NSC administration; the authors described that an intrathecal administration of neural stem cells, 3 days after CCI injury in rat, was able to significantly attenuate mechanical and thermal hyperalgesia with a marked increase of protein and mRNA levels of glial cell line derived neurotrophic factor (GDNF) in the spinal dorsal horn and dorsal root ganglia (DRG). So far we have considered the use of NSC for treating NP which follows a peripheral lesion of the nervous system; however, neural progenitors/stem cells were also used to treat lesions at spinal cord level. One of the main problems concerning their use in these conditions is represented by their low survival in the host damaged spinal cord. For this reason combinatorial strategies were developed to try to improve their transplant efficiency but the final outcome on NP is questionable. Positive results on pain were obtained by the group of Luo [22] investigating the efficacy of a cotransplantation of NSC and OECs (olfactory ensheathing cells) in a rat spinal cord transection injury model. They found that the transplantation of NSC together with OEC could improve the sensory function to mechanical and thermal stimuli after SCI; the authors suggested that OECs can promote the NSC survival and the cotransplantation downregulates the expression of NGF. Karimi-Abdolrezaee et al. [23] instead developed a combinatorial strategy that allows the successful application of neural progenitor cells (NPC) based therapies for the treatment of chronic spinal cord injury. The authors showed that chondroitin sulfate proteoglycans (CSPGs) in the glial scar around the site of chronic SCI negatively influences the long-term survival and integration of transplanted NPC and their therapeutic potential. For this reason they targeted CSPGs and one week later treated the same rats with transplants of NPC and transient infusion of growth factors (EGF, bFGF, and PDGF-AA). This combinatorial approach markedly increased the long-term survival of NPC and greatly optimized their migration and integration in the chronically injured spinal cord. Furthermore, this combined strategy promoted the axonal integrity and plasticity of the corticospinal tract and enhanced the plasticity of descending serotonergic pathways. These neuroanatomical changes were also associated with significantly improved neurobehavioral recovery after chronic SCI. However, cells were unable to modify the development of allodynia which follows the thoracic spinal cord injury. It is important to report that the first papers trying stem cell approaches in SCI models described negative results for pain relief. Hofstetter and colleagues [24] suggested a correlation between induction of allodynia after SCI and the transplantation of NPC. They reported that transplanted naive NPCs primarily differentiate into astrocytes and this was associated with induced aberrant sprouting of Calcitonin gene related peptide fibers rostral to the injury, leading to increased allodynia. In the same years, Macias et al. described that NSC primarily differentiated into astrocytes when transplanted into the injured spinal cord which resulted in thermal and mechanical forelimb allodynia [25].

All the papers mentioned above described the use of neural precursors/stem cells isolated from rodents; in literature, to our knowledge, there is only one paper which showed the results of using human neural stem cells in experimental animal models of NP. In this paper human neural stem cells are shown to be capable of surviving and differentiating in a traumatically injured environment improving the locomotor recovery [26]. However, in experimental paradigms of other pathologies, human neural stem cells (hNSC) have revealed anti-inflammatory and therapeutic abilities analogous to their murine counterpart [27–29]. Moreover, the possibility to isolate and expand hNSC lines of clinical grade [18] has allowed evaluating the safety of these cells in a phase I clinical trial in amyotrophic lateral sclerosis patients, which is currently underway.

## 3. Mesenchymal Stem Cells (MSC)

MSC are a heterogeneous subset of stromal stem cells which can be isolated from different sources: bone marrow [30], umbilical cord (UC) [31, 32], placenta [33], adipose tissue [34], dental pulp [35], and even the fetal liver [36] and lungs [37]. These cells express typical surface markers such as CD73, CD44, CD90, and CD105. Among MSC, the most representative ones are bone marrow MSC (BMSC), purified from bone marrow, and adipose tissue derived MSC (ASC), isolated from adipose tissue. ASCs are described to be BMSC migrated into the adipose tissue; hence there are no marked phenotypic differences between these two cell types [34, 38]. However, in recent years, other types of MSC, such as those derived from umbilical cord blood (UCB-MSC) and amniotic mesenchymal stem cells, have begun to attract researchers' attention for their therapeutic use.

A basic description of bone marrow may help clarify the origin of bone marrow derived mesenchymal stem cells. Bone marrow consists of a hematopoietic component (parenchyma) and a vascular component (stroma). The parenchyma includes hematopoietic stem cells and hematopoietic progenitor cells while bone marrow stroma contains multipotent nonhematopoietic progenitor cells, bone marrow stromal cells (MSC) that are known as multipotent cells capable of differentiating under specific experimental conditions into several types of cells, for example, osteoblasts, chondrocytes, adipocytes, and myocytes [30]. Moreover, some papers described the capacity of MSC to transdifferentiate also into neurons or astrocytes [11–13]. Both rodent and human MSC and bone marrow mononuclear cells were used for treating experimental neuropathic pain.

### 3.1. Bone Marrow MSC (BMSC)

#### 3.1.1. Rodent BMSC

One of the first groups to assess the effect of rat bone marrow stromal cells in an experimental rat model of peripheral neuropathy was the group of Musolino [39]. They demonstrated that an ipsilateral intraganglionic injection of rat bone marrow stromal cells was able to prevent the generation of mechanical allodynia and to reduce the number of allodynic responses to cold stimuli in rats that underwent a single ligature sciatic nerve constriction [39]. One of the possible mechanisms involved in such effect was the capacity of BMSC to partially prevent the injury-induced changes in galanin, Neuropeptide Y and Neuropeptide Y receptor expression in DRG [40]. The authors compared the effect of MSC on pain relief and biochemical changes to that of bone marrow nonadherent mononuclear cells (BNMCs), but these latter stem cells were, in that case, unable to reduce pain [39].

Rat bone marrow MSC has also been used in another type of neuropathic pain treatment, not derived from a direct nerve lesion, but consequence of the metabolic dysfunction present in diabetes which is one of the main causes of painful neuropathy in human. Shibata and colleagues tried in fact to improve diabetic polyneuropathy induced in rat by using Streptozotocin (STZ) [41]. MSC (1 × 10^6^) were therapeutically injected into the hind limb muscle 8 weeks after diabetes induction. The authors described an increase in VEGF and bFGF mRNA expression in MSC-injected diabetic rats and colocalized VEGF and bFGF in MSC in the transplanted site thus suggesting that MSC are responsible for growth factors secretion at the injected site. MSC were able to ameliorate all the alterations induced by diabetes such as hypoalgesia, delayed nerve conduction velocity, and decreased sciatic nerve blood flow. Moreover, MSC transplantation was able to normalize sural nerve morphometry restoring the axonal circularity, decreased in diabetic rats. The same positive effect on nerve conduction velocity amelioration was also reported by Kim and Jin [42], using the same model of diabetic neuropathy in mice, by injecting murine MSC into the hind limb muscle percutaneously along the course of the sciatic nerve at 4 sites. The improvement in nerve conduction velocity was attributed to the ability of MSC to increase trophic factors specific for neuronal populations in the PNS such as nerve growth factor (NGF) and neurotrophin-3 (NT-3). The authors did not directly assess pain.

#### 3.1.2. h(Human)BMSC

The Maione's group is the main user of human BMSC for treating experimental neuropathic pain. The authors use, as model of NP, the spared nerve injury (SNI) model in mice and administer hBMSC therapeutically, that is, 4 days after the surgery, injecting them either in the mouse lateral cerebral ventricle [43] or systemically into the caudal vein [44]. When intravenously injected, cells were able to home into the spinal cord and prefrontal cortex of SNI neuropathic mice. In both papers, hBMSC reduced pain-like behaviors, such as mechanical allodynia and thermal hyperalgesia, with an effect which was evident one week after cell transplantation and was long lasting. Indeed, when cells were injected into the caudal vein, their effect on pain relief was still present three months after transplant. The authors described the capacity of these cells to reduce glial [43] and macrophage activation [44] switching to an anti-inflammatory phenotype by decreasing the proinflammatory cytokines (IL-1 beta and IL-17) and increasing the anti-inflammatory cytokine IL-10 [43, 44].

The group of Waterman [45] developed a method to optimize the anti-inflammatory effects of human bone marrow MSC, skewing them in vitro, before their injections, towards a protective MSC2 phenotype. These MSC demonstrated a higher capacity to counteract mechanical allodynia and heat hypoalgesia induced in mice by STZ treatment. These cells were also able to decrease the serum level of proinflammatory cytokines and were described to be safe.

### 3.2. Adipose Tissue Derived MSC (ASC)

**Figure 1:**
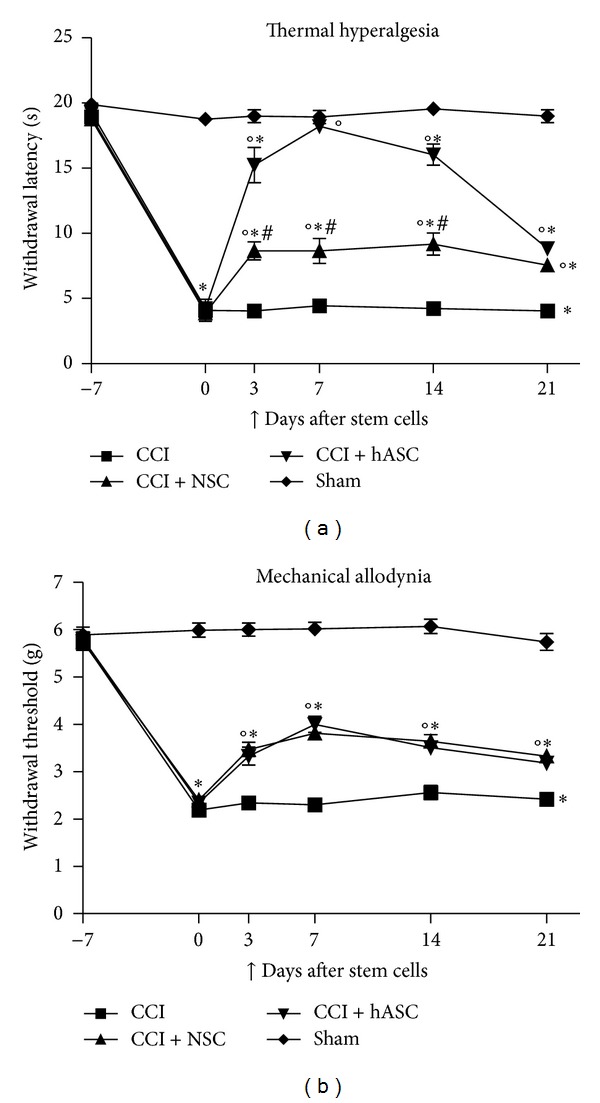
Time course of the effect of murine neural stem cells (NSCs) and human adipose derived stem cells (hASC) on thermal hyperalgesia (a), measured by Plantar test, and mechanical allodynia, measured by Dynamic Plantar Aesthesiometer (b), in neuropathic mice. 1 × 10^6^ NSCs/ASCs were injected intravenously 7 days after mice chronic constriction injury; their effect on pain was measured 3, 7, 14, and 21 days after the administration. Data represent mean +/− SEM of 7 mice. The statistical analysis was performed by using the two-way ANOVA analysis of variance followed by Bonferroni test. *P < 0.001 versus Sham, °*P* < 0.001 versus CCI, and ^#^ *P* < 0.001 versus hASC.

The great advantage of these cells, over the other kinds of MSC, is given by the possibility of isolating them by using low invasive procedures. These cells are in fact located in mature subcutaneous adipose tissue and can be obtained as litter of the fatty tissue after liposuction; the use of this tissue allows to obtain a large amount of MSC thus reducing, in some cases, the need of ex vivo culturing, leading eventually to lower the risk of developing chromosomal abnormalities due to the culture itself. Moreover, these cells are characterized by low immunogenicity and by high immunomodulatory properties which make them suitable for treating diseases in which the neuroinflammatory component plays a crucial role, such as NP. Not least these cells might be easily used for autologous transplant. Despite the high potential of these cells, their use for experimental neuropathic pain treatment is still limited. Our paper, recently published [46], was the first to assess the antinociceptive effect of hASC isolated from human adipose tissue of female donors undergoing plastic surgery. This paper is a complete work in which safety, antinociceptive effects, and biochemical changes induced by these cells were assessed. hASC were in vitro expanded [47, 48] and, after karyotype assessment, were injected into the caudal vein of neuropathic mice (CCI mice). Cells were injected, with a therapeutic intent, seven days after the surgery, in presence of a fully developed thermal hyperalgesia and mechanical allodynia. We clearly demonstrated a rapid, long lasting, and dose dependent antihyperalgesic and antiallodynic effect which could be reestablished with a second dose of cells when it began to vanish. The intravenous injection of 1 × 10^6^ hASCs was able to completely abolish thermal hyperalgesia starting one day after the injection [46]. The effects of hASCs on thermal hyperalgesia seem to be more potent than those of NSC [10]. In fact, as shown in Figure 1(a), the withdrawal thresholds of hASC treated mice were overall higher than those of NSC treated mice, and 7 days after hASC injection thermal hyperalgesia was completely abolished, while, for allodynia, a comparable effect of the two cells is evident (Figure 1(b)). The effect on pain relief well correlates with a general systemic and injured nerve localized anti-inflammatory effect of hASC. In fact, a significant increase of IL-10 serum concentration is already evident 1 day after hASC treatment; moreover at nerve site, the protein levels of IL-1, increased by the pathology, appeared normalized 1 day after the hASC injection, while the anti-inflammatory cytokine IL-10, decreased by CCI, gradually increased until reaching levels 3 times higher over control group [46]. The dose response effect, described for pain, was also evident for cytokines, indicating a clear correlation between pain relief and anti-inflammatory effect of hASCs. If we compare the effect on cytokines of hASC versus NSC, it is clear that the big difference between these two cell types regard their effect on IL-10. No changes at nerve site on IL-10 protein is evident seven days after NSC injection while, at the same time, IL-10 is strongly increased by hASC [46]. We assume that this effect, together with the general systemic anti-inflammatory one, could be responsible of the stronger antihyperalgesic effect of hASC. Besides the effects induced by hASC at nerve site we described also a normalization of the spinal cord iNOS protein level which is evident with a full neuropathic pain recovery. This paper clearly suggests a possible therapeutic use of hASC for neuropathic pain treatment.

These same cells and hATSCs, human adipose tissue-derived stem cells treated in vitro with ZnO shell nanoparticles in order to improve stem cell function, were recently used by In Choi et al. [49]; these cells, intrathecally injected, were able to reduce the pain consequent to a spinal cord injury by increasing the paw withdrawal thresholds to mechanical and thermal stimuli.

### 3.3. Umbilical Cord-Derived Mesenchymal Stem Cells (UC-MSC)

Human umbilical cord (UC) is a promising source of mesenchymal stem cells (MSC) and is nowadays under researchers' investigation. UC contains two umbilical arteries (UCAs) and one umbilical vein (UCV), both embedded within a specific mucous connective tissue, known as Wharton's jelly (WJ), which is covered by amniotic epithelium. MSC can be isolated from all these compartments by using different techniques; today it is still unclear which one is the best compartment in UC for clinical use. UC-MSC possess a gene expression profile similar to that of embryonic stem cells, but their collection procedure is considered ethically correct, and they are characterized by a faster self-renewal rate than MSC isolated, for example, from bone marrow. Moreover they have other attractive advantages which are summarized here: (1) a noninvasive collection procedure for autologous or allogeneic use; (2) a lower risk of infection; (3) a low risk of developing teratoma; (4) multipotency, and (5) low immunogenicity with a good immunosuppressive ability [50]. Roh and colleagues [51] recently investigated the therapeutic effect of transplanting human umbilical cord blood-derived mesenchymal stem cells (hUCB-MSC) or amniotic epithelial stem cells (hAESCs) on SCI-induced mechanical allodynia and thermal hyperalgesia in T13 spinal cord hemisected rats. Two weeks after SCI, hUCB-MSC or hAESC were transplanted around the spinal cord lesion site, and behavioral tests were performed; moreover, immunohistochemical and Western blot analyses were performed to evaluate possible therapeutic effects on SCI-induced inflammation and the nociceptive-related phosphorylation of the NMDA NR1 receptor subunit. The authors described only a weak antiallodynic effect of hUCB-MSC if compared to that of hAESCs and no effect on thermal hyperalgesia of either cell type. The antiallodynic effect of hAESCs is associated with a decrease in spinal cord microglia activity and NMDA receptor NR1 phosphorylation. In contrast to the weak efficacy of hUCB-MSC on pain symptoms, the group of Yang [52] using HUMSCs from Wharton's jelly of the umbilical cord transplanted into the spinal cord described a beneficial effect for wound healing and locomotor recovery after spinal cord injury in rats suggesting a potential use of these cells if not for pain at least for motor recovery.

## 4. Bone Marrow Derived Mononuclear Cells

An improvement in experimental neuropathic pain treatment was also obtained using other types of cells isolated from bone marrow and in particular by using bone marrow derived mononuclear cells. A paper of Klass et al. [53] described that the infusion (1 × 10^7^, i.v.) of rat marrow mononuclear cells, containing mixed stem cell populations, 10 days after rat CCI, was able to induce neuropathic pain recovery (both hyperalgesia and allodynia). The authors did not investigate into the mechanisms involved in such modulations. Freshly isolated rat bone marrow-derived mononuclear cells (BM-MNCs) were also used for contrasting diabetes neuropathy induced in rats by STZ [54]. Cells injected into the hind limb skeletal muscles two weeks after STZ were able to ameliorate mechanical hyperalgesia and cold allodynia in the BM-MNC-injected side. Furthermore, the slowed sciatic nerve conduction velocities (MNCV/SNCV) and decreased sciatic nerve blood flow in diabetic rats were improved in the BM-MNC-injected side. BM-MNC transplantation further decreased mRNA expression of NT-3 and number of microvessels in the hind limb.

## 5. Conclusions

In recent years, the possibility to apply stem cells for the treatment of neuropathic pain has attracted much attention, as demonstrated by the increasing number of preclinical studies in the literature (Table 1).

In whole the preclinical data here reported suggest positive effects of stem cells for relieving experimental neuropathic pain. An interesting point that emerges from the detailed analysis of the preclinical data is that peripheral neuropathic pain seems to be more responsive to stem cell treatment than pain arising from central lesion such as spinal cord injury. Moreover in SCI, stem cell treatment is not always able to positively and contemporarily affect both pain symptoms and motor recovery, indicating that different mechanisms can underlie the different effects.

It is important to underline that one of the main aspects concerning stem cells usage is both their fast onset and long lasting effect on pain relief; a single administration of cells is in fact able to induce an antiallodynic and antihyperalgesic effect which persists for long time, as it is still present up to 90 days after injection [44]. Generally, the conventional [8] and the newer pharmacological strategies [55, 56] for neuropathic pain treatment need a chronic treatment to be effective. The analgesic success of the commonly available drugs is often limited by side effects that appear increasing the administration dose or by the development of tolerance [8]. Moreover, in order to successfully approach this type of pain, patients often are treated with a combination of drugs with different mechanisms of action, increasing the risk of drug interaction and often reducing patient's compliance [9]. A more long lasting effect for some type of neuropathic pain such as low back pain or disk herniation can eventually be achieved by surgical approaches or epidural treatment, obviously exposing the patients to all the risks of the surgery. The clamorous effect of stem cells on pain relief in the preclinical tests may be related to their capacity to not only control pain as a symptom, but to act as disease modifier on the mechanisms at the basis of the development and maintenance of pain condition, for example, modulating the neuroimmune component which plays a relevant role in neuropathic pain. Despite these positive and encouraging considerations, there are many issues that need to be addressed and solved for a successful clinical translation. These points are well summarized in the review by Bonfield and Caplan [57] and include the classification of the cells, their efficacy and potency, their mode of administration, their dosage and their source, together with the final goal of the analysis, and the tracking of the stem cell. Among these, as emerged in this review, the route of administration of stem cells represents an important variable which may also influence the choice of the final number of cells to be injected. Strategies for local stem cell delivery can be applied to the treatment of well localized lesions but are, however, described to increase risks and side effects such as bleeding and tissue injury [58]; certainly, from a clinical point of view, a systemic delivery is attractive, given the broad biodistribution and easy access. On the other hand, we have to point out that this route is, in some cases, associated with a passive cell entrapment within tissues that do not represent the main target of treatment, which may potentially lead to unwanted effects and may be eventually associated to a reduced effect of the cells. The homing of stem cells after a systemic injection represents in fact a much debated topic. In our first paper we described the capacity of stem cells to specifically reach the damaged nerve [10]. Although we observed a low transplant efficiency, we did not find the cells into other critical tissues such as lungs. Also Maione's group [44] reported the ability of MSC to home central nervous system areas critically involved in NP signaling describing only a scarce presence of stem cell in the lungs. In general, however, other papers report a marked lung first passage effect of the cells which limits the number of cells which can reach the area of injury [59–61]. Overall the literature agrees with the general idea that stem cells, even in a limited number, can interact with the host cells and orchestrate a long lasting modulation resulting, most of the times, in a final beneficial therapeutic effect [10, 44, 58]. Another strictly related question is the toxicity and the possible malignant transformation and cytogenetic aberrations of stem cells. The literature quite agrees on the safety of stem cells [62, 63] but by a careful analysis of the preclinical papers reported here, it emerges that this aspect has not been specifically or adequately considered. In our work [46] we injected different doses of hASC reaching the highest dose of 6 × 10^6^ cells/mice and we did not register any macroscopic adverse effect: no animal died or changed its habits/behaviour and no side effects have been observed. The safety of a similar dose of hASC intravenously infused in animals and humans was also described by Ra and colleagues [64]; the authors did not register any side effect or tumor mass formation in the three months after cell infusion. Also the paper by Waterman et al. [45] described no premature mortality or morbidity due to MSC treatment and the necropsy of the cell treated animals revealed no macroscopic pathology of any of the major organs. In contrast, Djouad and colleagues [65] described an increase of tumor formation in animals likely due to the immunosuppressive effects of MSC, rather than to a direct transformation of stem cells in tumor cells. Even though, as discussed, there are still many open points that need better understanding, a clear trend to clinical use of stem cells also in treating pain is apparent, as demonstrated by a very recent and scientifically sound paper [66] that reported a preliminary human study in which the autologous administration of adipose derived stem cells in the facial tissue was able to attenuate orofacial neuropathic pain symptoms. The cells were injected perineurally directly into the center of origin of pain and in the adjacent pain field of the affected branches of the trigeminal nerve. The effect of the treatment was evident 6 months after cell injection and cells were described to be safe, well tolerated by the patients, and accompanied by a significant reduction of analgesic drug doses. What is clear is that the research on stem cells is evolving; newly discovered populations of stem cells begin to be characterized and used in the regenerative medicine. The bioactive molecules that can be released by these same stem cells are starting to be identified and are likely effectors/candidates for the therapeutic effect. As an example the beneficial role of the medium conditioned by MSC for improving motor recovery was recently described [67]. Finally several reports indicate that the regenerative [68] and immunomodulatory [69] effects of MSC can be partially reproduced by the microvesicles (MVs) that are shed by activated MSC and that can be isolated from their culture medium [69]. On the basis of these considerations it is to be expected that the panorama of neuropathic pain treatment will change again shortly.

## Figures and Tables

**Table 1:** Stem cells used for experimental neuropathic pain treatment.

Cell source	Delivery site	Number of cells	Model of NP and species	Effect on pain	Author and year
Neural stem cells					
NSC (mouse)	Intravenous	1, 2, 3 × 10^6^	CCI (mouse)	Improvement of thermal hyperalgesia and mechanical allodynia	Franchi et al., 2012 [10]
NSC (rat)	Intrathecal	1 × 10^6^	CCI (rat)	Improvement of thermal and mechanical hyperalgesia	Xu et al., 2013 [21]
NSC + OEC (rat)	Injury site	3 × 10^5^	SCI (rat)	Cotransplantation improves sensory function	Luo et al., 2013 [22]
NPC (mouse)	Injury site	4 × 10^5^	SCI (rat)	No effect on pain (allodynia)	Karimi-Abdolrezaee et al., 2010 [23]
NPC (rat)	Injury site	1 × 10^5^	SCI (rat)	Induction of allodynia	Hofstetter et al., 2005 [24]
NSC (mouse)	Injury site	1 × 10^5^	SCI (rat)	Induction of thermal and mechanical forelimb allodynia	Macias et al., 2006 [25]
Bone marrow mesenchymal stem cells					
MSC (rat)	Intraganglionic (DRG)	2 × 10^5^	SLNC (rat)	Prevention of mechanical and thermal allodynia	Musolino et al., 2007 [39]
MSC (rat)	Injection in the hind limb skeletal muscle	1 × 10^6^	STZ-induced diabetes (rat)	Improvement of hypoalgesia	Shibata et al., 2008 [41]
MSC (human)	Lateral cerebral ventricle	5 × 10^4^	SNI (mouse)	Improvement of mechanical allodynia and thermal hyperalgesia	Siniscalco et al., 2010 [43]
MSC (human)	Intravenous	2 × 10^6^	SNI (mouse)	Improvement of thermal hyperalgesia and mechanical allodynia	Siniscalco et al., 2011 [44]
MSC2 (human)	Intraperitoneal	5 × 10^5^, 1 × 10^6^	STZ-induced diabetes (mouse)	Improvement of mechanical allodynia and heat hypoalgesia	Waterman et al., 2012 [45]
Adipose tissue derived-mesenchymal stem cells					
ASC (human)	Intravenous	5 × 10^5^, 1 × 10^6^	CCI (mouse)	Improvement of thermal hyperalgesia and mechanical allodynia	Sacerdote et al., 2013 [46]
ATSC/core shell particle-treated ATSC (human)	Intrathecal	Not indicated	SCI (mouse)	Improvement of mechanical allodynia and thermal hyperalgesia	In Choi et al., 2013 [49]
Umbilical cord mesenchymal stem cells					
UCB-MSC (human)	Injury site	1 × 10^6^	SCI (rat)	Tendency to reduce mechanical allodynia	Roh et al., 2013 [51]
(HUMSCs) isolated from Wharton's jelly (human)	Injury site	5 × 10^5^	SCI (rat)	Locomotor recovery	Yang et al., 2008 [52]
Bone marrow derived mononuclear cells					
Marrow mononuclear cells (rat)	Intravenous	1 × 10^7^	CCI (rat)	Improvement of thermal and mechanical allodynia	Klass et al., 2007 [53]
BM-MNC (rat)	Injected into 10 points in the unilateral femoral quadriceps, femoral biceps, and soleus muscles	1 × 10^6^	STZ-induced diabetes (rat)	Improvement of mechanical and thermal allodynia	Naruse et al., 2011 [54]
